# A Curious Custom in Chili

**Published:** 1892-12

**Authors:** 


### A CURIOUS CUSTOM IN CHILI.

In this queer country there is a funny side even to funerals. The other day a sound of music attracted me to the window, and what do you think I saw? A mahogany hued peon (peasant) carrying on his head and outstretched hands a plank about five feet long and on the plank a dead child. The little corpse was that of a girl, apparently about five years old. It was attired in a short frock of red calico, the legs incased in coarse white hose " a world too wide for the shrunk shank ;" no shoes, the jet black hair smoothly braided and crowned by a jaunty wreath of paper roses and the cheeks horribly daubed with vermillion to simulate the hue of health. The plank bearer was closely followed by two women, evidently the mother and grandmother of the deceased, and they walked with an air of conscious importance, as becomes those who have furnished *otra angelta* (" another little angel," as here a dead child is universally called) to swell the heavenly host. Behind the women marched two men, playing with might and main, one on a fiddle, the other on a guitar, each intent on a tune of his own regardless of the other's performance ; and the rear brought up by a dozen or more laughing and chattering men, women and children, most of whom gave indubitable evidence of unwise generosity on somebody's part in the way of *chicha*, the Chilian low class intoxicant.

They were on the way to the Pantheno to inter the " little angel," over whom they had been dancing for several days, and which possibly had been loaned once or twice inthe meantime to friends who were not so fortunate as to have a corpse in the family. Among the more degraded class of Chilians it is the general custom to make death an excuse for orgies wild and ridiculous, and the body, especially of a child, is kept for festive purposes until it becomes offensive to people passing the house.

